# Effective coverage measurements and cascade for maternal, newborn, child and adolescent health in high-income countries: systematic review

**DOI:** 10.7189/jogh.15.04178

**Published:** 2025-06-20

**Authors:** Marianna Zanette, Georgia Konstantinou, Josephine Exley, Debra Jackson, Marzia Lazzerini, ‘Life Stage Quality of Care Metrics Technical Working Group (LSQCM TWG) for Maternal, Newborn, Child and Adolescent Health and Ageing – subgroup on Effective Coverage’, Ambrose Agweyu, Emily Carter, Rowan Harwood, Kathleen Hill, Shogo Kubota, Hannah H Leslie, Abdoulaye Maiga, Tanya Marchant, Andrew D Marsh, Kim Minjoon, Muzigaba Moise, Jean-Pierre Monet, Melinda Munos, Alicia Quach, Sara Riese, Florina Serbanescu, Ashley L Sheffel, Kathleen L Strong, Ahmed Tashrik, Jr Nuhu Omeiza Yaqub

**Affiliations:** 1 Institute for Maternal and Child Health IRCCS Burlo Garofolo, World Health Organization (WHO) Collaborating Centre (CC) for Maternal and Child Health, Trieste, Italy; 2 London School of Hygiene & Tropical Medicine, London, UK; 3 London School of Hygiene & Tropical Medicine, Health Services Research and Policy, London, UK; 4 London School of Hygiene & Tropical Medicine, Maternal, Adolescent, Reproductive & Child Health (MARCH) Centre, London, UK; 5 University of the Western Cape, School of Public Health, Cape Town, South Africa; 6 London School of Hygiene & Tropical Medicine, Disease Control, London, UK

## Abstract

**Background:**

The concept of ‘effective coverage’ (EC) aims to combine the concept of coverage with the quality of care delivered and, ultimately, the health benefits received by the population in need. To date, systematic reviews of EC of maternal, newborn, child and adolescent health (MNCAH) have focused on low- and middle-income countries (LMICs). No review has examined whether and how the concept has been applied in high-income countries (HICs). To address this gap, this systematic review investigated the application of EC measures in MNCAH care in HICs.

**Methods:**

This was a systematic review that followed the Preferred Reporting Items for Systematic reviews and Meta-Analyses (PRISMA) reporting guidelines. The search strategy was developed from previous EC reviews conducted in LMICs and further adapted to the HIC setting. Additional search terms were identified through discussion with experts from the Life Stage Quality of Care Metrics Technical Working Group subgroup on EC. We searched three databases, PubMed, Embase, and Web of Science, over 10 years. We conducted additional searches in Google Scholar and by consulting members of the Life Stage Quality of Care Metrics Technical Working Group. We did not pose any language or type of article limits.

**Results:**

The database search identified 18 976 studies for screening. Of these, 672 abstracts were screened, and none of the full texts considered met our inclusion criteria (*e.g.* human immunodeficiency virus/hepatitis c virus continuum of care cascade, intervention type, qualitative search-interviews/questionnaire type studies). Thirty-two articles were retrieved through the additional search strategies, and none were included because of LMIC-focused research. Therefore, examples of EC of MNCAH care applied in HICs were not identified.

**Conclusions:**

Further investigation should be conducted into the application of the EC concept for assessing MNCAH care in HICs. This research will help us understand how this concept can be used to support health system effectiveness, efficiency, and equity in HICs.

**Registration:**

The study protocol was registered at the Open Science Framework: https://doi.org/10.17605/OSF.IO/FMCG8.

Global efforts to track improvements in maternal, newborn, child and adolescent health (MNCAH) have focused on metrics that capture individuals’ ‘contact with’ or ‘access to’ the health system or ‘number of people receiving’ a health intervention. However, evidence demonstrates that indicators that only capture the steps related to service contact or receipt of health interventions tend to overestimate the health benefits as they take no account of the quality of care received [1]. Health systems need more comprehensive measurements for tracking the performance of health services and interventions and for effectively planning health policies to address observed gaps [2].

Quality of care is a multi-dimensional concept and has been defined in terms of the inputs necessary to deliver a health service or intervention, the care process delivered, including the provision of care according to national/international standards/guidelines, and the outcomes achieved [3]. Effective coverage (EC) aims to move beyond service contact or intervention coverage by also capturing the quality of care delivered and, ultimately, the health benefit received by the population in need [2] and has been recommended by the World Health Organization (WHO) and the Lancet Global Health Commission on High-Quality Health Systems as a preferred measure to assess health system performance [4].

The WHO and United Nations International Children's Emergency Fund (UNICEF) established the ‘EC Think Tank Group’ in 2019 to establish standardised definitions and measurement approaches of EC for MNCAH [5]. The group defined EC as ‘the proportion of a population in need of a health service that had a positive health outcome from the service’ [5,6] and recommended EC to be described using a health-service coverage cascade. This cascade includes six steps that the target population needs to move through to achieve a positive outcome (**Figure 1**). As a comprehensive measure, EC can be used to estimate the impact of the interventions carried out by the health system on people's health [2]. The concept of EC is relevant both in low-and middle-income countries (LMICs) and high-income countries (HICs).

**Figure 1:**
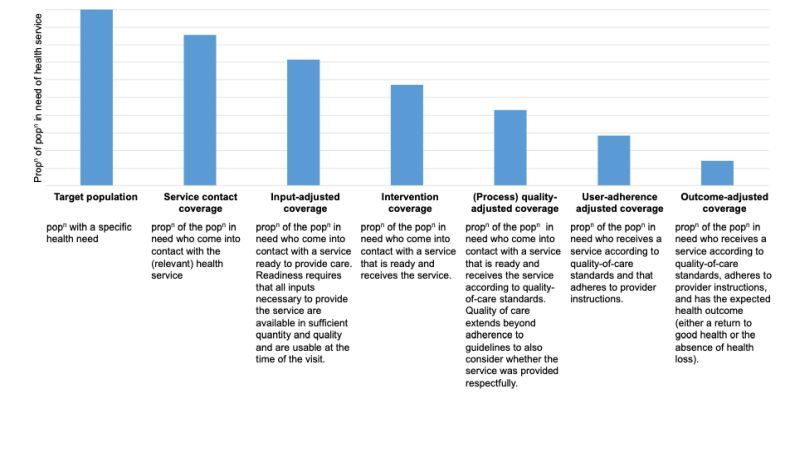
Health service coverage cascade for measuring effective coverage (from Exley et al. 2022, adapted from Marsh et al. 2020) [6]. Pop^n^ – population, Prop^n^ – proportion.

So far, the literature reports a few systematic reviews on the use of the EC cascade for assessing maternal, newborn, child and adolescent health and nutrition services in LMICs [1,2,6,7], while no review has yet focused on the application of the EC cascade in HICs. This systematic review aimed at synthesising the existing evidence on the application of EC measures in MNCAH care in HICs. This review is part of an extensive literature review, including the study of the EC measures in maternal-neonatal-child health care in LMICs (unpublished study) [7].

## METHODS

The review aimed to identify and synthetise studies conducted in HICs that measured EC of MNCAH care interventions and/or services. The systematic review followed standardised methods and was reported according to the PRISMA guidelines [8]. The PRISMA checklist is reported in Table S1 in the **Online Supplementary Document**. The study protocol was registered at the Open Science Framework: https://doi.org/10.17605/OSF.IO/FMCG8.

### Eligibility criteria

**Table 1** reports the inclusion and exclusion criteria. Briefly, studies were eligible for inclusion if they examined any MNCAH intervention or service in a HIC and the outcome reported was either explicitly described as an EC measure (or relevant synonym, eg, quality-adjusted coverage) or if they defined the target population and accounted for at least two additional steps of the EC cascade, at least one of them capturing the quality of care provided. No restrictions on the study design were used.

**Table 1:** Inclusion and exclusion criteria for the literature review screening process

Variables	Inclusion criteria	Exclusion criteria
Population	Studies that defined the appropriate target population in need of a health service or intervention.	Studies that did not define and quantify the population in need.
	Studies conducted among women during pregnancy and childbirth, newborns, children and adolescent.	Studies including also adults, in which data on the MNCA population are not reported separately
Interventions	Any MNCAH health services.	
Outcome measures	Any study that presented methods used to measure a population-level adjusted contact coverage measure.	Studies that do not provide sufficient detail on the items used to construct the effective coverage measure in the paper, appendices, or other supplementary documents.
	Studies needed to: 1) explicitly state measuring Effective Coverage/Quality-adjusted Coverage/Outcome-Adjusted Coverage or 2) define appropriate population in need AND combine minimum two additional components of the remaining EC cascade steps, and at least one of them capturing the quality of care provided	
Study design	Any publication or data source to estimate Effective Coverage	
	Abstracts and conference presentations if enough data are presented to determine how the EC measure was constructed	
Language	Any language	
Setting	Studies conducted in HICs	Studies conducted in LMICs
	Studies conducted in health facilities, communities, home and schools	
Time	Studies published from 2013	

EC – effective coverage, HICs – high-income countries, LMICs – low-and middle-income countries, MNCA – maternal neonatal child adolescent, MNCAH – maternal neonatal child adolescent health

### Search strategy

The search strategy was developed in subsequent steps. First, the search strategy was identified from previous EC reviews conducted in LMICs, and the search terms were adapted and extended with additional terms according to HIC settings; for the EC concept, all the synonyms listed in the previous literature reviews *(e.g.* quality-adjusted coverage, continuum of care, *etc*.) were employed. Second, the list of LMICs was substituted with the list of all HICs based on the World Bank classification [9]; third, the list of search terms was further expanded, following the recommendations of experts from the ‘Life Stage Quality of Care Metrics (LSQCM) Technical Working Group (TWG) for Maternal, Newborn, Child and Adolescent Health and Ageing’ (LSQCM-TWG) subgroup on EC, as displayed in Table S2–4 in the **Online Supplementary Document**. All search terms were in English, but no other language restrictions were applied to the searches and articles screened.

Three databases, PubMed, Embase, and Web of Science, were searched from 13 November 2013 to 13 November 2023. This large timeframe was chosen according to the results of the existing literature reviews and the LSQCM-TWG expert consultation. Additional searches were conducted in Google Scholar over the same timeframe using the function **‘**cited by**’** to identify studies that had cited the previous reviews on the EC concept, and consulting members of the LSQCM TWG to ensure that no relevant studies were missed.

### Selection process

After duplicate removal, the title and abstract were screened by one reviewer (MZ). A second reviewer screened 15% of the titles (GK) and 50% of the abstracts (JE). Two reviewers independently screened the full texts (JE and MZ). The independent screening process reached a high level of agreement between two reviewers. Any discrepancies between reviewers were discussed with the other co-authors until a common consensus was reached. In case of disagreement/uncertainty among the co-authors, such as in the case of the human immunodeficiency virus (HIV) care cascade [10,11], the discussion was extended to the members of the TWG.

Endnote 20 (EndNote, Clarivate, Philadelphia, PA, USA) was used to manage retrieved references and eliminate duplicates. For the screening process, the references were entered into Excel (Microsoft Inc, Seattle, WA, USA). The Excel database included information on authors, year of publication, title and abstract.

## RESULTS

**Figure 2:**
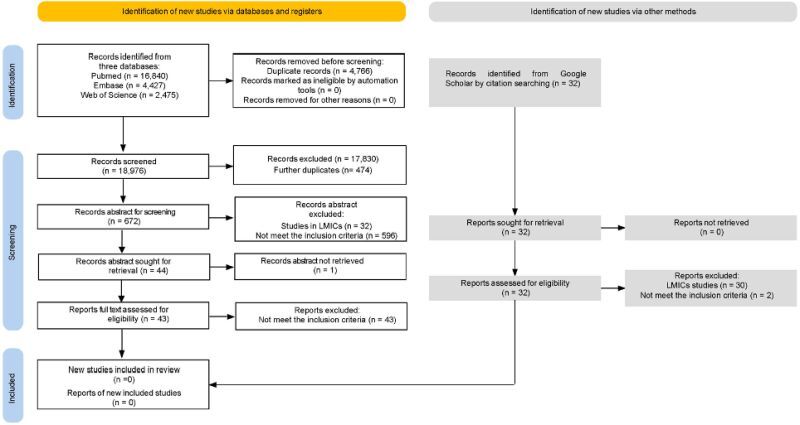
PRISMA flow diagram (2020 version for new systematic reviews). EC – effective coverage, LMICs – low- and middle-income countries, PRISMA –Preferred Reporting Items for Systematic reviews and Meta-Analyses.

After removing duplicates, the database search identified 18 976 records for screening (**Figure 2**). Of these, 672 titles were selected for abstract screening. Among these, 32 articles were excluded because they were conducted in LMICs, one abstract and related full text were not found, and 596 did not meet the inclusion criteria. Of the 43 full texts considered, none met our inclusion criteria. Thirty-two additional articles were retrieved through the additional searches in Google Scholar; 30 were excluded because they were conducted in LMICs, and the remaining two did not meet the inclusion criteria – the PRISMA flow diagram is reported in **Figure 2**. No examples of EC of MNCAH care were identified in HICs.

While no examples related to MNACH were identified during full-text screening, three studies that examined EC for health services/interventions in HICs fell outside this review's scope (*i.e*. eye care services, major depressive disorders, and severe mental health disorders) [12–14]. Two of these studies undertook cross-country comparisons and included LMICs and HICs, while the third was conducted in Italy alone. None of the studies cited or measured the EC as described by Marsh et al.

Further, among the full texts examined, two articles on HIV-infected youths in the USA described the HIV continuum of care cascade, which applies a similar stepwise approach to the EC cascade to describe the steps that an adolescent living with HIV needs to go through to achieve viral suppression (**Table 2**) [10,11]. Comparing the HIV care cascades against the EC cascade, both studies defined the target population: Lally et al. started with individuals with a known HIV infection, whereas Zanoni et al. identified a preceding step by estimating the number of people likely to be infected and only Zanoni et al. measured service contact. Neither measured inputs nor quality of care (QoC); both examined receipt of therapy (intervention coverage); only Zanoni et al. measured retention in care (user-adherence), and both examined outcomes (viral suppression). Both articles were excluded after discussion with the LSQM TWG members, as it was recognised that both described a different concept from EC (they expressed the ‘continuum of care cascade’, and not EC).

**Table 2:** The stepwise processes of the continuum of care cascade in HIV-infected youth in the USA as described by Zanoni et al. and Lally et al. articles [8,9]

Articles	Target population	Steps			
Zanoni et al. (2014)	Estimated number of HIV-infected individual aged 13–29 the USA	Diagnosed: have been diagnosed with HIV	Linked: have been linked to care	Retained: remain in care and maintain a high degree of adherence to ART	Outcome: individual supressed, those achieve viral suppression.
Lally et al. (2018)	Individuals behaviourally-infected with HIV aged 13–24 attending adolescent medicine clinics	Care engagement: had at least two HIV primary care visits	Linked: reported a health care provider prescribed ART		Outcome: individual attained suppression during the study period

ART – antiretroviral therapy, HIV – human immunodeficiency virus

## DISCUSSION

Previous reviews have examined the application of EC in LMICs but have not examined whether EC has been used in HICs [1,2,6,7]. This review sought to fill this gap by examining whether EC has been used to measure MNCAH in HICs; however, no example of EC in MNCAH care applied in HICs has been identified.

Although the EC concept is not entirely unknown to authors in HICs, as reported by the three articles on adult eye care and mental health disorders identified by this review [12–14], it appears not to have been applied in relation to MNCAH. None of the studies cited or captured the EC definition, despite being published after the EC Think Tank Group had published its recommendations for how to define EC.

Concerning the HIV continuum of care cascade papers excluded in this systematic review [8,9], it is acknowledged that the continuum of care cascade is a well-established concept that has also been applied to other infectious diseases, like tuberculosis, Hepatitis C, cancer and non-communicable diseases [15]. The continuum of care cascade and EC are different concepts, although they both share a step-wise approach. The continuum of care cascade focuses more on a patient’s perspective by tracking individual patients at each step, while EC aims to capture more broadly a health system perspective, evidenced by the inclusion of the required health systems inputs (health system readiness) and quality of care provided.

The lack of identified studies on EC of MNCAH in HICs may have possible explanations. Many HICs have well-established national policy frameworks with settled measures for QoC evaluation, like defined standards and QoC indicators inside standardised protocols and processes, guidelines, internal and external assessments, patient surveys, and the international accreditation process for health services. These measurements/processes aim at improving QoC, assessing the country’s health system on the safe and effective delivery of care. In particular, international accreditation represents a well-established process in many HICs. Its ultimate goals – improving health care services and health outcomes, ensuring QoC and safety, as defined by Shaw [16] – overlap with the goals of the EC cascade. From a historical perspective, it must be acknowledged that EC has been promoted mostly in LMICs, most notably by WHO and other international agencies. Its use in LMICs has benchmarked performance across countries and drove the Universal Health Coverage agenda [16,17]. The lack of traction in HICs may potentially reflect broader trends, with innovations on MNCAH metrics being adopted in LMICs but not in HICs.

In LMICs, over 50 studies have been reported by systematic reviews on EC, providing examples of how EC has been operationalised for different health services/health conditions [1,2,6,7]. The application of EC in LMICs showed some limitations in the area of investigation – often focused on antenatal and intrapartum care and care for frequent childhood conditions like fever, diarrhoea, and pneumonia – and challenges in the EC application concept [1,2,6,7]. The challenges that many LMICs have faced in measuring EC, such as the lack of routine data to measure process quality without defined QoC standards for many health services/interventions [15], or user adherence and outcomes, which can be complex and expensive to measure for some health settings/conditions [2,15], can likely be easier to overcome in many HICs, where there are established health information management systems and frequent routine patient surveys.

Despite the lack of studies serving as a model for future research on EC in HICs, and despite the fact that in HICs, there are often large data health systems in place, as well as standards of QoC and evaluation processes, EC could be a valuable tool for supporting improvements in the quality of MNCAH services in HICs. For instance, in some HICs, where a large amount of data are routinely collected in the health information management system, the EC cascade may help with evaluating and improving the QoC in a defined and standardised way, both at the national health system and MNCAH care services levels. In areas where data collection is less well-established, the EC cascade can provide a well-defined and step-wise QoC evaluation and improvement process. In both cases, more research is needed to understand how to operationalise EC in the setting of HICs.

Although rigorous methods were followed, some limitations, like including only three large databases, might have led to missing relevant evidence, and all pertinent literature might not have been captured, given the considerable variation in terminology used [6]. However, to minimise these limitations, the search terms included articles that did not explicitly mention EC using a wide range of synonyms. Additional searches on Google Scholar and consultation with experts were undertaken. In addition, no restrictions on the language of the articles screened or the type of study were posed. All these decisions resulted in a high number of papers screened.

## CONCLUSIONS

The application of the EC concept for assessing MNCAH care in HICs, as well as its relevance and utility, should be further investigated. Further research is needed to understand how this concept can be used to support health system effectiveness, efficiency, and equity in HICs and how it can be incorporated into the quality improvement researchers' and policymakers' agendas.

## Additional material


Online Supplementary Document
