# Supplementary material for: Effective coverage measurements and cascade for maternal, newborn, child and adolescent health in high-income countries: systematic review
Source: J Glob Health. 2025 Jun 20;15:04178. doi: 10.7189/jogh.15.04178 (PMC12180102; doi:10.7189/jogh.15.04178)
Supplement: Online Supplementary Document [file jogh-15-04178-s001.pdf]

Supplement to: Zanette M, Konstantinou G, Exley J, Jackson, Lazzerini M; Life Stage Quality of Care Metrics Technical Working Group (LSQCM TWG) for Maternal, Newborn, Child and Adolescent Health and Ageing – subgroup on Effective Coverage. Effective coverage measurements and cascade for maternal, newborn, child and adolescent health in high-income countries: systematic review. J Glob Health. 2025;15:04178.

Table S1. PRISMA 2020 literature review checklist.

Section and Topic	Item #	Checklist item	Location where item is reported
**TITLE**			
Title	1	Identify the report as a systematic review.	Title article, (page 1)
**ABSTRACT**			
Abstract	2	See the PRISMA 2020 for Abstracts checklist.	Abstract article, (page 2)
**INTRODUCTION**			
Rationale	3	Describe the rationale for the review in the context of existing knowledge.	First three paragraphs of the Introduction
Objectives	4	Provide an explicit statement of the objective(s) or question(s) the review addresses.	Last paragraph of the Introduction
**METHODS**			
Eligibility criteria	5	Specify the inclusion and exclusion criteria for the review and how studies were grouped for the syntheses.	"Eligibility criteria" paragraph of Methods, Table 1
Information sources	6	Specify all databases, registers, websites, organisations, reference lists and other sources searched or consulted to identify studies. Specify the date when each source was last searched or consulted.	"Search strategy" paragraph of Methods
Search strategy	7	Present the full search strategies for all databases, registers and websites, including any filters and limits used.	Appendix S1 in the Online Supplementary Document.
Selection process	8	Specify the methods used to decide whether a study met the inclusion criteria of the review, including how many reviewers screened each record and each report retrieved, whether they worked independently, and if applicable, details of automation tools used in the process.	"Eligibility criteria" and "Selection process" paragraphs of Methods
Data collection process	9	Specify the methods used to collect data from reports, including how many reviewers collected data from each report, whether they worked independently, any processes for obtaining or confirming data from study investigators, and if applicable, details of automation tools used in the process.	"Selection process" paragraph of Methods
Data items	10a	List and define all outcomes for which data were sought. Specify whether all results that were compatible with each outcome domain in each study were sought (e.g. for all measures, time points, analyses), and if not, the methods used to decide which results to collect.	"Selection process" paragraph of Methods
	10b	List and define all other variables for which data were sought (e.g. participant and intervention characteristics, funding sources). Describe any assumptions made about any missing or unclear information.	Not applicable
Study risk of bias assessment	11	Specify the methods used to assess risk of bias in the included studies, including details of the tool(s) used, how many reviewers assessed each study and whether they worked independently, and if applicable, details of automation tools used in the process.	"Selection process" paragraph of Methods
Effect measures	12	Specify for each outcome the effect measure(s) (e.g. risk ratio, mean difference) used in the synthesis or presentation of results.	Not applicable
Synthesis methods	13a	Describe the processes used to decide which studies were eligible for each synthesis (e.g. tabulating the study intervention characteristics and comparing against the planned groups for each synthesis (item #5)).	"Eligibility criteria" and "Selection process" paragraphs of Methods
	13b	Describe any methods required to prepare the data for presentation or synthesis, such as handling of missing summary statistics, or data conversions.	Not applicable
	13c	Describe any methods used to tabulate or visually display results of individual studies and syntheses.	Figure 1, Table 1, "Eligibility criteria" and "Selection process" paragraph of Methods
	13d	Describe any methods used to synthesize results and provide a rationale for the choice(s). If meta-analysis was performed, describe the model(s), method(s) to identify the presence and extent of statistical heterogeneity, and software package(s) used.	Not applicable
	13e	Describe any methods used to explore possible causes of heterogeneity among study results (e.g. subgroup analysis, meta-regression).	Not applicable
	13f	Describe any sensitivity analyses conducted to assess robustness of the synthesized results.	Not applicable

Section and Topic	Item #	Checklist item	Location where item is reported
Reporting bias assessment	14	Describe any methods used to assess risk of bias due to missing results in a synthesis (arising from reporting biases).	Not applicable
Certainty assessment	15	Describe any methods used to assess certainty (or confidence) in the body of evidence for an outcome.	"Selection process" paragraph of Methods
**RESULTS**			
Study selection	16a	Describe the results of the search and selection process, from the number of records identified in the search to the number of studies included in the review, ideally using a flow diagram.	First paragraph of the Results and Figure 2
	16b	Cite studies that might appear to meet the inclusion criteria, but which were excluded, and explain why they were excluded.	Second and third paragraphs of the Results and Table 2
Study characteristics	17	Cite each included study and present its characteristics.	Not applicable
Risk of bias in studies	18	Present assessments of risk of bias for each included study.	Not applicable
Results of individual studies	19	For all outcomes, present, for each study: (a) summary statistics for each group (where appropriate) and (b) an effect estimate and its precision (e.g. confidence/credible interval), ideally using structured tables or plots.	Not applicable
Results of syntheses	20a	For each synthesis, briefly summarise the characteristics and risk of bias among contributing studies.	Not applicable
	20b	Present results of all statistical syntheses conducted. If meta-analysis was done, present for each the summary estimate and its precision (e.g. confidence/credible interval) and measures of statistical heterogeneity. If comparing groups, describe the direction of the effect.	Not applicable
	20c	Present results of all investigations of possible causes of heterogeneity among study results.	Not applicable
	20d	Present results of all sensitivity analyses conducted to assess the robustness of the synthesized results.	Not applicable
Reporting biases	21	Present assessments of risk of bias due to missing results (arising from reporting biases) for each synthesis assessed.	Not applicable
Certainty of evidence	22	Present assessments of certainty (or confidence) in the body of evidence for each outcome assessed.	Not applicable
**DISCUSSION**			
Discussion	23a	Provide a general interpretation of the results in the context of other evidence.	The first six paragraphs of the Discussion
	23b	Discuss any limitations of the evidence included in the review.	The last paragraph of the Discussion
	23c	Discuss any limitations of the review processes used.	The last paragraph of the Discussion
	23d	Discuss implications of the results for practice, policy, and future research.	Conclusion
**OTHER INFORMATION**			
Registration and protocol	24a	Provide registration information for the review, including register name and registration number, or state that the review was not registered.	First paragraph of the Methods
	24b	Indicate where the review protocol can be accessed, or state that a protocol was not prepared.	Not applicable
	24c	Describe and explain any amendments to information provided at registration or in the protocol.	Not applicable
Support	25	Describe sources of financial or non-financial support for the review, and the role of the funders or sponsors in the review.	"Acknowledgements" and "Funding" sections
Competing interests	26	Declare any competing interests of review authors.	"Disclosure of interest" section
Availability of data, code and other materials	27	Report which of the following are publicly available and where they can be found: template data collection forms; data extracted from included studies; data used for all analyses; analytic code; any other materials used in the review.	"Data availability" section

From: Page MJ, McKenzie JE, Bossuyt PM, Boutron I, Hoffmann TC, Mulrow CD, et al. The PRISMA 2020 statement: an updated guideline for reporting systematic reviews. BMJ 2021;372:n71. doi: 10.1136/bmj.n71.

Table S2. Search strategy in PubMed

Term	Concept	Search terms 13.11.23 PubMed (limits Human and 10 years)	Results
1	Effective Coverage	"effective coverage" or "structural qualit*" or "process* quality" or "service* quality" or "care-seeking pathway*" or "content coverage" or "continuum of care" or "health services coverage cascade*" or "effective cascade coverage " or "quality-adjusted coverage" or "intervention coverage" or "input-adjusted coverage" or "user-adherence coverage" or "outcome-adjusted coverage" or "intervention* quality-adjusted coverage" or "input* intervention* coverage" or "user-adherence input*" or "treatment pathway*" or "obstetric service* coverage" or "facility readiness" or "high-quality contact*" or "missed opportunit*" or "quality care population access" or "high-quality adequate contact"	**53,213**
2	Mothers	"maternal mortality" or "maternal morbidity" or "maternal death*" or "maternal safety" or "maternal complication*" or "maternal emergenc*" or "maternal care" or "maternal health-care" or "maternal health" or "maternal outcome*" or "maternal labo?r care" or "maternal delivery" or "maternal health service*" or "mother*" or "obstetric* mortality" or "obstetric* morbidity" or "obstetric* death*" or "obstetric* safety" or "obstetric* complication*" or "obstetric* emergenc*" or "obstetric* care" or "obstetric* healthcare" or "obstetric* health" or "obstetric outcome*" or "obstetric* labo?r care" or "obstetric* health service*" or "motherhood" or "childbirth" or "RMNCH" or "MNCH" or "MNH" or "facility delivery" or "facility birth" or "facility-based birth" or "institutional birth" or "birth" or "intrapartum" or "perinatal" or "peripartum" or "postpartum" or "postnatal" or "prenatal" or "antenatal" or "antepartum" or "postpartum" or "preconception" or "PPC" or "pregnancy complication*" or "cesarian section" or "pregnancy" or "maternal welfare" or "maternal hospital*" or "wom?n health*" or "wom?n care" or "wom?n health service*" or "wom?n welfare" or "obstetric* delivery"	**393,275**
3	Newborn	"new-born*" or "new-born mortality" or "new-born morbidity" or "new-born death" or "new-born complication" or "new-born safety" or "new-born emergenc*" or "new-born outcome" or "new-born care" or "new-born healthcare" or "new-born health" or "new-born health service" or "new-born service" or "new-born hospital" or "new-born health-centre" or "neonatal mortality" or "neonatal morbidity" or "neonatal death" or "neonatal complication" or "neonatal safety" or "neonatal emergenc*" or "neonatal outcome" or "neonatal care" or "neonatal health-care" or "neonatal health" or "neonatal health-service" or "neonatal service" or "neonatal hospital" or "neonatal health-centre" or "f?etal mortality" or "f?etal death" or "f?etal complications" or "f?etal safety" or "f?etal emergenc*" or "f?etal outcome" or "f?etal care" or "f?etal healthcare" or "f?etal health" or "still-born" or "stillbirth" or "birth" or "birth care" or "birth service" or "birth health-care" or "birth complication" or "f?etus" or "childbirth mortality" or "childbirth morbidity" or "childbirth complication" or "childbirth care" or "childbirth health service" or "childbirth service" or "childbirth hospital" or "baby" or "babies" or "infant*" or "neonate*" or "young infant*" or "newborn illness" or "breastfeeding" or "infant feeding" or "maternal-child health intervention" or "MCH intervention" or "infant mortality" or "fetal disease" or "neonatal intensive care units"	**437,685**
4	Child	"child*" or "child mortality" or "child morbidity" or "child death" or "child complication" or "child safety" or "child emergenc*" or "child outcome" or "child care" or "child healthcare" or "child health" or "child health service" or "child hospital" or "child illness" or "child sickness" or "child disease" or "child care seeking" or "child health treatment" or "child therap*" or "child HIV" or "child immune?ation" or "child nutrition" or "child cancer" or "juvenile mortality" or "juvenile morbidity" or "juvenile death" or "juvenile complication" or "juvenile safety" or "juvenile emergenc*" or "juvenile outcome" or "juvenile care" or "juvenile healthcare" or "juvenile health" or "juvenile health service" or "juvenile illness" or "juvenile sickness" or "juvenile disease" or "juvenile care seeking" or "juvenile health treatment" or "juvenile therap*" or "juvenile HIV" or "juvenile immune?ation" or "juvenile nutrition" or "juvenile cancer" or "pre-school mortality" or "pre-school morbidity" or "pre-school death" or "toddler*" or "kid" or "kids" or "boy" or "boys" or "girl" or "girls" or "childhood"	**1,030,091**

5	**Adolescent**	"adolescent*" or "adolescent maternity" or "adolescent pregnancy" or "adolescent contraception" or "adolescent friendly health-centre" or "friendly health-centre" or "adolescent friendly care" or "friendly care" or "adolescent mortality" or "adolescent morbidity" or "adolescent death" or "adolescent safety" or "adolescent complication" or "adolescent emergenc*" or "adolescent care" or "adolescent health-care" or "adolescent health" or "adolescent outcome" or "adolescent antenatal care" or "adolescent postnatal care" or "adolescent postpartum care" or "adolescent health service" or "adolescent continuum of care" or "continuum of care" or "adolescent illness*" or "adolescent sickness" or "adolescent disease" or "adolescent care seeking" or "adolescent health treatment" or "adolescent therapy" or "adolescent HIV" or "adolescent immune?ation" or "adolescent nutrition" or "adolescent cancer" or "teen maternity" or "teen pregnancy" or "teen contraception" or "teen friendly health-centre" or "teen friendly care" or "teen mortality" or "teen morbidity" or "teen death" or "teen safety" or "teen complication" or "teen emergenc*" or "teen care" or "teen healthcare" or "teen health" or "teen outcome" or "teen antenatal care" or "teen postnatal care" or "teen postpartum care" or "teen health-service" or "teen friendly care" or "teen friendly healthcare" or "teen contraception" or "teen continuum of care" or "teen illness" or "teen sickness" or "teen disease" or "teen care seeking" or "teen health treatment" or "teen therapy" or "teen HIV" or "teen immune?ation" or "teen nutrition" or "teen cancer" or "teenager*"	**677,524**
6	**Population**	#2 OR #3 OR #4 OR#5	**1,585,109**
7	**Setting**	American Samoa or Andorra or Antigua or Barbuda or Aruba or Australia or Austria or Bahamas or Bahrain or Barbados or Belgium or Bermuda or "British Virgin Islands" or Brunei or Bulgaria or Canada or "Cayman Islands" or "Channel Islands" or Chile or Croatia or Curacao or Cyprus or "Czech Republic" or Denmark or Estonia or "Faroe Islands" or Finland or France or "French Polynesia" or Germany or Gibraltar or Greece or Greenland or Guam or Guyana or "Hong Kong" or Hungary or Iceland or Ireland or "Isle of Man" or Israel or Italy or Japan or "Korea Republic" or Kuwait or Latvia or Liechtenstein or Lithuania or Luxembourg or Macau or Malta or Monaco or Nauru or Netherlands or "New Caledonia" or "New Zealand" or "Northern Mariana Islands" or Norway or Oman or Palau or Panama or Poland or Portugal or Puerto Rico or Qatar or Romania or Saint Kitts and Nevis or Saint Martin or "San Marino" or "Russian federation" or "Saudi Arabia" or Seychelles or Singapore or "Sint Maarten" or Slovakia or Slovenia or "South Korea" or Spain or Sweden or Switzerland or Taiwan or Trinidad or Tobago or Turks or "Caicos Islands" or "United Arab Emirates" or "United Kingdom" or "United States" or "Virgin Islands" or Uruguay or "rich* countr*" or "Western countr*" or "European countr*" or "North America" or Europe or "more developed nation*" or "developed world" or "wealthy countr*" or "rich* nation*" or "upper econom*" or "rich* econom*" or "high*-income econom*" or "wealth econom*" or "developed econom*" or "more advanced countr*" or "Industriali?ed state*" or "Industrially advanced countr*" or "high*-income nation*" or "high*-developed economies" or "industriali?ed societies" or "developed countr*" or "developed world econom*" or "economic countr*" or "rich countr*" or "wealthy nation*" or "affluent countr*" or "commodity rich countr*" or "affluent nation*" or "Western nation*" or "European nation*" or "North America nation*" or "high-developed nation*" or "industriali?ed nation*"	**4,073,159**
8	**Final Search**	1 AND 6 AND 7	**16,384**
9	**Additional search terms with the same limit and timeframe**	"gestational diabetes" or "GDM" or "pre-eclampsia" or "eclampsia" or "post-partum haemorrhage" or "antepartum haemorrhage" or "antepartum bleeding" "placentae abruption" or "uterus rupture" or "gestational cholestasis" or "obstructed labour" or "prolonged labour" or "chorioamnionitis" or "maternal obesity" or "gestational hypothyroidism" or "gestational hyperthyroidism" or "gestational cancer" or "mola" or "trophoblast* cancer" or "maternal mental health" or "maternal well-being" or "respectful maternal care" or "preterm labo?r" or "preterm delivery" or "preterm newborn*" or "prematurity" or "small for gestational age" or "SGA" or "macrosomia" or "intrauterine growth retardation" or "IUGR" or "IUFD" or "intrauterine f?etal demise" or "f?etal abnormalit*" or "f?etal malformations" or "f?etal genetic disease*" or "neonatal diarrhea" or "neonatal diarrhoea" or "neonatal fever" or "neonatal infection" or "neonatal sepsis" or "neonatal respiratory distress syndrome" or "neonatal brain haemorrhage" or "neonatal jaundice" or "neonatal pneumonia" or "neonatal-HIV-transmission" or "neonatal hypoglycaemia" or "neonatal vaccination" or "neonatal retinopathy" or "new-born diarrhea" or "new-born diarrhoea" or "new-born fever" or "new-born infection" or "new-born sepsis" or "new-born respiratory distress syndrome" or "new-born brain haemorrhage" or "new-born jaundice" or "new-born pneumonia" or "new-born-HIV-transmission" or "new-born hypoglycaemia" or "new-born vaccination" or "new-born retinopathy" or "child diarrhea" or "child diarrhoea" or "child fever" or "child infection" or "child sepsis" or "child jaundice" or "child pneumonia" or "child-HIV-transmission" or "child diabetes" or "child vaccination" or "child autoimmune disease" or "child obesity" or "child malnutrition"	**78,597**
10	**Additional results**	1 AND 7 AND 9	**472**

Table S3. Search strategy in Embase

Term	Concept	EMBASE Search terms 13.11.23 (limits human and 10 years applied at the final search)	Results
1	Effective Coverage	“effective coverage” or “structural qualit*” or “process* quality “ or “service* quality” or “care-seeking pathway*” or “content coverage” or “continuum of care” or “health services coverage cascade*” or “effective cascade coverage” or “quality-adjusted coverage” or “intervention coverage” or “input-adjusted coverage” or “user-adherence coverage” or “outcome-adjusted coverage” or “intervention* quality-adjusted coverage” or “input* intervention* coverage” or “user-adherence input*” or “treatment pathway*” or “obstetric service* coverage” or “facility readiness“ or “high-quality contact*” or “missed opportunit*” or “quality care population access“ or “high-quality adequate contact”	36,940
2	Mothers	“maternal mortality” or “maternal morbidity” or “maternal death*” or “maternal safety” or “maternal complication*” or “maternal emergenc*” or “maternal care” or “maternal health-care” or “maternal health” or “maternal outcome*” or “maternal labo?r care” or “maternal delivery” or “maternal health service*” or “mother*” or “obstetric* mortality” or “obstetric* morbidity” or “obstetric* death*” or “obstetric* safety” or “obstetric* complication*” or “obstetric* emergenc*” or “obstetric* care” or “obstetric* healthcare” or “obstetric* health” or “obstetric* outcome*” or “obstetric* labo?r care” or “obstetric* health service*” or “motherhood” or “childbirth” or “RMNCH” or “MNCH” or “MNH” or “facility delivery” or “facility birth” or “facility-based birth” or “institutional birth” or “birth” or “intrapartum” or “perinatal” or “peripartum” or “postpartum” or “postnatal” or “prenatal” or “antenatal” or “antepartum” or “postpartum” or “preconception” or “PPC” or “pregnancy complication*” or “cesarian section” or “pregnancy” or “maternal welfare” or “maternal hospital*” or “wom?n health*” or “wom?n care” or “wom?n health service*” or “wom?n welfare” or “obstetric* delivery”	2,044,913
3	Newborn	“new-born*” or “new-born mortality” or “new-born morbidity” or “new-born deaths” or “new-born complication” or “new-born safety” or “new-born emergenc*” or “new-born outcome” or “new-born care” or “new-born healthcare” or “new-born health” or “new-born health service” or “new-born service” or “new-born hospital” or “new-born health-centre” or “neonatal mortality” or “neonatal morbidity” or “neonatal death” or “neonatal complication” or “neonatal safety” or “neonatal emergenc*” or “neonatal outcome” or “neonatal care” or “neonatal health-care” or “neonatal health” or “neonatal health-service” or “neonatal service” or “neonatal hospital” or “neonatal health-centre” or “f?etal mortality” or “f?etal death” or “f?etal complications” or “f?etal safety” or “f?etal emergenc*” or “f?etal outcome” or “f?etal care” or “f?etal healthcare” or “f?etal health” or “still-born” or “stillbirth” or “birth” or “birth care” or “birth service” or “birth health-care” or “birth complication” or “f?etus” or “childbirth” or “childbirth mortality” or “childbirth morbidity” or “childbirth complication” or “childbirth care” or “childbirth health service” or “childbirth service” or “childbirth hospital” or “baby” or “babies” or “infant*” or “neonate*” or “young infant*” or “newborn illness” or “breastfeeding” or “infant feeding” or “maternal-child health intervention” or “MCH intervention” or “infant mortality” or “fetal disease” or “neonatal intensive care units”	1,803,817
4	Child	“child*” or “child mortality” or “child morbidity” or “child death” or “child complication” or “child safety” or “child emergenc*” or “child outcome” or “child care” or “child healthcare” or “child health” or “child health service” or “child hospital” or “child illness” or “child sickness” or “child disease” or “child care seeking” or “child health treatment” or “child therap*” or “child HIV” or “child immune?ation” or “child nutrition” or “child cancer” or “juvenile mortality” or “juvenile morbidity” or “juvenile death” or “juvenile complication” or “juvenile safety” or “juvenile emergenc*” or “juvenile outcome” or “juvenile care” or “juvenile healthcare” or “juvenile health” or “juvenile health service” or “juvenile illness” or “juvenile sickness” or “juvenile disease” or “juvenile care seeking” or “juvenile health treatment” or “juvenile therap*” or “juvenile HIV” or “juvenile immune?ation” or “juvenile nutrition” or “juvenile cancer” or “pre-school mortality” or “pre-school morbidity” or “pre-school death” or “toddler*” or “kid” or “kids” or “boy” or “boys” or “girl” or “girls” or “childhood”	4,320,694

5	**Adolescent**	"adolescent*" or "adolescent maternity" or "adolescent pregnancy" or "adolescent contraception" or "adolescent friendly health-centre" or "adolescent friendly care" or "adolescent mortality" or "adolescent morbidity" or "adolescent death" or "adolescent safety" or "adolescent complication" or "adolescent emergenc*" or "adolescent care" or "adolescent health-care" or "adolescent health" or "adolescent outcome" or "adolescent antenatal care" or "adolescent postnatal care" or "adolescent postpartum care" or "adolescent health service" or "adolescent continuum of care" or "adolescent illness*" or "adolescent sickness" or "adolescent disease" or "adolescent care seeking" or "adolescent health treatment" or "adolescent therapy" or "adolescent HIV" or "adolescent immune?ation" or "adolescent nutrition" or "adolescent cancer" or "teen maternity" or "teen pregnancy" or "teen contraception" or "teen friendly health-centre" or "teen friendly care" or "teen mortality" or "teen morbidity" or "teen death" or "teen safety" or "teen complication" or "teen emergenc*" or "teen care" or "teen healthcare" or "teen health" or "teen outcome" or "teen antenatal care" or "teen postnatal care" or "teen postpartum care" or "teen health-service" or "teen friendly care" or "teen friendly healthcare" or "teen contraception" or "teen continuum of care" or "teen illness" or "teen sickness" or "teen disease" or "teen care seeking" or "teen health treatment" or "teen therapy" or "teen HIV" or "teen immune?ation" or "teen nutrition" or "teen cancer" or "teenager*"	**2,131,317**
6	**Population**	1 OR 2 OR 3 OR 4 OR 5	**6,771,816**
7	**Setting**	American Samoa or Andorra or Antigua or Barbuda or Aruba or Australia or Austria or Bahamas or Bahrain or Barbados or Belgium or Bermuda or "British Virgin Islands" or Brunei or Bulgaria or Canada or "Cayman Islands" or "Channel Islands" or Chile or Croatia or Curacao or Cyprus or "Czech Republic" or Denmark or Estonia or "Faroe Islands" or Finland or France or "French Polynesia" or Germany or Gibraltar or Greece or Greenland or Guam or Guyana or "Hong Kong" or Hungary or Iceland or Ireland or "Isle of Man" or Israel or Italy or Japan or "Korea Republic" or Kuwait or Latvia or Liechtenstein or Lithuania or Luxembourg or Macau or Malta or Monaco or Nauru or Netherlands or "New Caledonia" or "New Zealand" or "Northern Mariana Islands" or Norway or Oman or Palau or Panama or Poland or Portugal or Puerto Rico or Qatar or Romania or Saint Kitts and Nevis or Saint Martin or "San Marino" or "Russian federation" or "Saudi Arabia" or Seychelles or Singapore or "Sint Maarten" or Slovakia or Slovenia or "South Korea" or Spain or Sweden or Switzerland or Taiwan or Trinidad or Tobago or Turks or "Caicos Islands" or "United Arab Emirates" or "United Kingdom" or "United States" or "United States" or "Virgin Islands" or Uruguay or "rich* countr*" or "Western countr*" or "European countr*" or "North America" or Europe or "more developed nation*" or "developed world" or "wealthy countr*" or "rich* nation*" or "upper econom*" or "rich* econom*" or "high*-income econom*" or "wealth econom*" or "developed econom*" or "more advanced countr*" or "Industriali?ed state*" or "Industrially advanced countr*" or "high*-income nation*" or "high*-developed economies" or "industriali?ed societies" or "developed countr*" or "developed world econom*" or "economic countr*" or "rich countr*" or "wealthy nation*" or "affluent countr*" or "commodity rich countr*" or "affluent nation*" or "Western nation*" or "European nation*" or "North America nation*" or "high-developed nation*" or "industriali?ed nation*"	**30,546,073**
8	**Final Search**	1 AND 6 AND 7	**4,260**
9	**Additional search terms with the same limit and timeframe**	"gestational diabetes" or "GDM" or "pre-eclampsia" or "eclampsia" or "post-partum haemorrhage" or "antepartum haemorrhage" or "antepartum bleeding" "placentae abruption" or "uterus rupture" or "gestational cholestasis" or "obstructed labour" or "prolonged labour" or "chorioamnionitis" or "maternal obesity" or "gestational hypothyroidism" or "gestational hyperthyroidism" or "gestational cancer" or "mola" or "trophoblast* cancer" or "maternal mental health" or "maternal well-being" or "respectful maternal care" or "preterm labo?r" or "preterm delivery" or "preterm newborn*" or "prematurity" or "small for gestational age" or "SGA" or "macrosomia" or "intrauterine growth retardation" or "IUGR" or "IUFD" or "intrauterine f?etal demise" or "f?etal abnormalit*" or "f?etal malformations" or "f?etal genetic disease*" or "neonatal diarrhea" or "neonatal diarrhoea" or "neonatal fever" or "neonatal infection" or "neonatal sepsis" or "neonatal respiratory distress syndrome" or "neonatal brain haemorrhage" or "neonatal jaundice" or "neonatal pneumonia" or "neonatal-HIV-transmission" or "neonatal hypoglycaemia" or "neonatal vaccination" or "neonatal retinopathy" or "new-born diarrhea" or "new-born diarrhoea" or "new-born fever" or "new-born infection" or "new-born sepsis" or "new-born respiratory distress syndrome" or "new-born brain haemorrhage" or "new-born jaundice" or "new-born pneumonia" or "new-born-HIV-transmission" or "new-born hypoglycaemia" or "new-born vaccination" or "new-born retinopathy" or "child diarrhea" or "child diarrhoea" or "child fever" or "child infection" or "child sepsis" or "child jaundice" or "child pneumonia" or "child-HIV-transmission" or "child diabetes" or "child vaccination" or "child autoimmune disease" or "child obesity" or "child malnutrition"	**260,450**
10	**Additional results**	1 AND 7 AND 9	**167**

Table S4. Search strategy in Web of Science

Term	Concept	Web of Science Search terms 13.11.23 (limits Human and 10 years)	Results
1	Effective Coverage	“effective coverage” or “structural qualit*” or “process* quality “ or “service* quality” or “care-seeking pathway*” or “content coverage” or “continuum of care” or “health services coverage cascade*” or “effective cascade coverage” or “quality-adjusted coverage” or “intervention coverage” or “input-adjusted coverage” or “user-adherence coverage” or “outcome-adjusted coverage” or “intervention* quality-adjusted coverage” or “input* intervention* coverage” or “user-adherence input*” or “treatment pathway*” or “obstetric service* coverage” or “facility readiness“ or “high-quality contact*” or “missed opportunit*” or “quality care population access“ or “high-quality adequate contact”	**53,117**
2	Mothers	“maternal mortality” or “maternal morbidity” or “maternal death*” or “maternal safety” or “maternal complication*” or “maternal emergenc*” or “maternal care” or “maternal health-care” or “maternal health” or “maternal outcome*” or “maternal labo$r care” or “maternal delivery” or “maternal health service*” or “mother*” or “obstetric* mortality” or “obstetric* morbidity” or “obstetric* death*” or “obstetric* safety” or “obstetric* complication*” or “obstetric* emergenc*” or “obstetric* care” or “obstetric* healthcare” or “obstetric* health” or “obstetric outcome*” or “obstetric* labo$r care” or “obstetric* health service*” or “motherhood” or “childbirth” or “RMNCH” or “MNCH” or “MNH” or “facility delivery” or “facility birth” or “facility based birth” or “institutional birth” or “birth” or “intrapartum” or “perinatal” or “peripartum” or “postpartum” or “postnatal” or “prenatal” or “antenatal” or “antepartum” or “postpartum” or “preconception” or “PPC” or “pregnancy complication*” or “cesarian section” or “pregnancy” or “maternal welfare” or “maternal hospital*” or “wom$n health*” or “wom$n care” or “wom$n health service*” or “wom$n welfare” or “obstetric* delivery”	**678,116**
3	Newborn	“new-born*” or “new-born mortality” or “new-born morbidity” or “new-born deaths” or “new-born complication” or “new-born safety” or “new-born emergenc*” or “new-born outcome” or “new-born care” or “new-born healthcare” or “new-born health” or “new-born health service” or “new-born service” or “new-born hospital” or “new-born health-centre” or “neonatal mortality” or “neonatal morbidity” or “neonatal death” or “neonatal complication” or “neonatal safety” or “neonatal emergenc*” or “neonatal outcome” or “neonatal care” or “neonatal health-care” or “neonatal health” or “neonatal health-service” or “neonatal service” or “neonatal hospital” or “neonatal health-centre” or “f$etal mortality” or “f$etal death” or “f$etal complications” or “f$etal safety” or “f$etal emergenc*” or “f$etal outcome” or “f$etal care” or “f$etal healthcare” or “f$etal health” or “still-born” or “stillbirth” or “birth” or “birth care” or “birth service” or “birth health-care” or “birth complication” or “f$etus” or “childbirth” or “childbirth mortality” or “childbirth morbidity” or “childbirth complication” or “childbirth care” or “childbirth health service” or “childbirth service” or “childbirth hospital” or “baby” or “babies” or “infant*” or “neonate*” or “young infant*” or “newborn illness” or “breastfeeding” or “infant feeding” or “maternal-child health intervention” or “MCH intervention” or “infant mortality” or “fetal disease” or “neonatal intensive care units”	**547,990**
4	Child	“child*” or “child mortality” or “child morbidity” or “child death” or “child complication” or “child safety” or “child emergenc*” or “child outcome” or “child care” or “child healthcare” or “child health” or “child health service” or “child hospital” or “child illness” or “child sickness” or “child disease” or “child care seeking” or “child health treatment” or “child therap*” or “child HIV” or “child immune?ation” or “child nutrition” or “child cancer” or “juvenile mortality” or “juvenile morbidity” or “juvenile death” or “juvenile complication” or “juvenile safety” or “juvenile emergenc*” or “juvenile outcome” or “juvenile care” or “juvenile healthcare” or “juvenile health” or “juvenile health service” or “juvenile illness” or “juvenile sickness” or “juvenile disease” or “juvenile care seeking” or “juvenile health treatment” or “juvenile therap*” or “juvenile HIV” or “juvenile immune?ation” or “juvenile nutrition” or “juvenile cancer” or “pre-school mortality” or “pre-school morbidity” or “pre-school death” or “toddler*” or “kid” or “kids” or “boy” or “boys” or “girl” or “girls” or “childhood”	**1,800,994**
5	Adolescent	“adolescent*” or “adolescent maternity” or “adolescent pregnancy” or “adolescent contraception” or “adolescent friendly health-centre” or “adolescent friendly care” or “adolescent mortality” or “adolescent morbidity” or “adolescent death” or “adolescent safety” or “adolescent complication” or “adolescent emergenc*” or “adolescent care” or “adolescent health-care” or “adolescent health” or “adolescent outcome” or “adolescent antenatal care” or “adolescent postnatal care” or “adolescent postpartum care” or “adolescent health service” or “adolescent continuum of care” or “adolescent illness*” or “adolescent sickness” or “adolescent disease” or “adolescent care seeking” or “adolescent health treatment” or “adolescent therapy” or “adolescent HIV” or “adolescent immune?ation” or “adolescent nutrition” or “adolescent cancer” or “teen maternity” or “teen pregnancy” or “teen contraception” or “teen friendly health-centre” or “teen friendly care” or “teen mortality” or “teen morbidity” or “teen death” or “teen safety” or “teen complication” or “teen emergenc*” or “teen care” or “teen healthcare” or “teen health” or “teen outcome” or “teen antenatal care” or “teen postnatal care” or “teen postpartum care” or “teen health-service” or “teen friendly care” or “teen friendly healthcare” or “teen contraception” or “teen continuum of care” or “teen illness” or “teen sickness” or “teen disease” or “teen care	**409,649**

		seeking” or “teen health treatment” or “teen therapy” or “teen HIV” or “teen immune?ation” or “teen nutrition” or “teen cancer” or “teenager*”	
**6**	**Population**	1 OR 2 OR 3 OR 4 OR 5	**2,416,897**
**7**	**Setting**	American Samoa or Andorra or Antigua or Barbuda or Aruba or Australia or Austria or Bahamas or Bahrain or Barbados or Belgium or Bermuda or “British Virgin Islands” or Brunei or Bulgaria or Canada or “Cayman Islands” or “Channel Islands” or Chile or Croatia or Curacao or Cyprus or “Czech Republic” or Denmark or Estonia or “Faroe Islands” or Finland or France or “French Polynesia” or Germany or Gibraltar or Greece or Greenland or Guam or Guyana or “Hong Kong” or Hungary or Iceland or Ireland or “Isle of Man” or Israel or Italy or Japan or “Korea Republic” or Kuwait or Latvia or Liechtenstein or Lithuania or Luxembourg or Macau or Malta or Monaco or Nauru or Netherlands or “New Caledonia” or “New Zealand” or “Northern Mariana Islands” or Norway or Oman or Palau or Panama or Poland or Portugal or Puerto Rico or Qatar or Romania or Saint Kitts and Nevis or Saint Martin or “San Marino” or “Russian federation” or “Saudi Arabia” or Seychelles or Singapore or “Sint Maarten” or Slovakia or Slovenia or “South Korea” or Spain or Sweden or Switzerland or Taiwan or Trinidad or Tobago or Turks or “Caicos Islands” or “United Arab Emirates” or “United Kingdom” or “United States” or “United States” or “Virgin Islands” or Uruguay or “rich* countr*” or “Western countr*” or “European countr*” or “North America” or Europe or “more developed nation*” or “developed world” or “wealthy countr*” or “rich* nation*” or “upper econom*” or “rich* econom*” or “high*-income econom*” or “wealth econom*” or “developed econom*” or “more advanced countr*” or “Industriali?ed state*” or “Industrially advanced countr*” or “high*-income nation*” or “high*-developed economies” or “industriali?ed societies” or “developed countr*” or “developed world econom*” or “economic countr*” or “rich countr*” or “wealthy nation*” or “affluent countr*” or “commodity rich countr*” or “affluent nation*” or “commodity rich countr*” or “affluent nation*” or “Western nation*” or “European nation*” or “North America nation*” or “high-developed nation*” or “industriali?ed nation*”	**16,714,625**
**8**	**Final Search**	1 AND 6 AND 7	**2,442**
**9**	**Additional search terms with the same limit and timeframe**	“gestational diabetes” or “GDM” or “pre-eclampsia” or “eclampsia” or “post-partum haemorrhage” or “antepartum haemorrhage” or “antepartum bleeding” “placentae abruption” or “uterus rupture” or “gestational cholestasis” or “obstructed labour” or “prolonged labour” or “chorioamnionitis” or “maternal obesity” or “gestational hypothyroidism” or “gestational hyperthyroidism” or “gestational cancer” or “mola” or “trophoblast* cancer” or “maternal mental health” or “maternal well-being” or “respectful maternal care” or “preterm labo$r” or “preterm delivery” or “preterm newborn*” or “prematurity” or “small for gestational age” or “SGA” or “macrosomia” or “intrauterine growth retardation” or “IUGR” or “IUFD” or “intrauterine f$etal demise” or “f$etal abnormalit*” or “f$etal malformations” or “f$etal genetic disease*” or “neonatal diarrhea” or “neonatal diarrhoea” or “neonatal fever” or “neonatal infection” or “neonatal sepsis” or “neonatal respiratory distress syndrome” or “neonatal brain haemorrhage” or “neonatal jaundice” or “neonatal pneumonia” or “neonatal-HIV-transmission” or “neonatal hypoglycaemia” or “neonatal vaccination” or “neonatal retinopathy” or “new-born diarrhea” or “new-born diarrhoea” or “new-born fever” or “new-born infection” or “new-born sepsis” or “new-born respiratory distress syndrome” or “new-born brain haemorrhage” or “new-born jaundice” or “new-born pneumonia” or “new-born-HIV-transmission” or “new-born hypoglycaemia” or “new-born vaccination” or “new-born retinopathy” or “child diarrhea” or “child diarrhoea” or “child fever” or “child infection” or “child sepsis” or “child jaundice” or “child pneumonia” or “child-HIV-transmission” or “child diabetes” or “child vaccination” or “child autoimmune disease” or “child obesity” or “child malnutrition”	**94,788**
**10**	**Additional results**	1 AND 7 AND 9	**33**
